# Characterization of a New Silk Sericin-Based Hydrogel for Water Retention in Soil

**DOI:** 10.3390/polym15132763

**Published:** 2023-06-21

**Authors:** Natalia Jaramillo-Quiceno, Catalina Álvarez-López, Gustavo Adolfo Hincapié-Llanos, Carlos A. Hincapié, Marisol Osorio

**Affiliations:** 1 Grupo de Investigación Sobre Nuevos Materiales, Universidad Pontificia Bolivariana, Medellín 050031, Colombia; catalina.alvarezl@upb.du.co; 2 Grupo de Investigaciones Agroindustriales (GRAIN), Universidad Pontificia Bolivariana, Medellín 050031, Colombia; gustavo.hincapie@upb.edu.co (G.A.H.-L.); carlos.hincapie@upb.edu.co (C.A.H.); 3 Grupo de Investigación en Gestión de la Tecnología y la Innovación (GTI), Universidad Pontificia Bolivariana, Medellín 050031, Colombia; marisol.osorio@upb.edu.co

**Keywords:** sericin, hydrogel, water retention, transfer function, data-driven-models, MATLAB

## Abstract

Hydrogel-type absorbent materials are currently a technological alternative for improving water retention in the soil and reducing nutrient loss by leaching and evaporation. This study aimed to evaluate the application of a new hydrogel based on silk sericin (SS) as a water retention material in soil. The morphology of the hydrogel was characterized using Scanning Electron Microscopy (SEM), and its impact on moisture retention in sandy loam soil (SLS) under different levels of matric pressure (MP) was evaluated. Additionally, water content data were collected over time for both SLS and SLS with hydrogel (SLS + H), and the data were used to fit predictive models. The results indicate that the hydrogel had a porous morphology that promoted water retention and soil release. Under a MP of 0.3 bar, the use of the hydrogel increased water retention by 44.70% with respect to that of SLS. The predictive models developed were adequately adjusted to the behavior of the moisture data over time and evidenced the incidence of the absorbent material on the dynamics of the moisture content in the soil. Therefore, these models could be useful for facilitating subsequent simulations or for designing automatic soil moisture control systems oriented to smart farming.

## 1. Introduction

It is estimated that by 2050, the world population will approach 10 billion inhabitants [1]. This trend implies that the demand for food will continue to grow, and to meet this demand, it is estimated that it will be necessary to increase crop efficiency worldwide by approximately 70% and, in the case of developing countries, by up to 100% [2,3]. Thus, the pressure exerted on renewable water resources will increase and soil fertility will need to be artificially improved, which, in accordance with the traditional agricultural production system, would imply an increase in the use of NPK fertilizers composed of three primary macronutrients: nitrogen (N), phosphorus (P), and potassium (K). Such an increase would have a much more significant impact on nitrogen and phosphorus use. In most crops, the efficiency of nitrogen fertilizers is sonly 40% [4], whereas only 16% of the total phosphorus extracted worldwide is used for human consumption [5]. This implies an important economic and environmental problem for the future, since, under current fertilizer application models, a large proportion of fertilizers are lost through leaching, volatilization, evaporation, and/or degradation, causing cost overruns [6], soil degradation, increased global warming [2,7], eutrophication of water sources, and, in some cases, phytotoxicity [8].

An alternative to mitigate the problems mentioned above is the development of absorbent materials, such as hydrogels. These materials consist of three-dimensional polymeric networks that can absorb and retain large amounts of water (up to a thousand times their dry weight) without dissolving [9]. The presence of hydrophilic segments or groups in these materials confers them a high affinity for water, whereas the stability and flexibility of their structures are due to the crosslinking of the polymeric chains that constitute them [6,10]. Recently, hydrogels have been evaluated for several agriculture applications, and it was demonstrated that they allow keeping the soil moisture for extended periods and, at the same time, regulating the slower release of agrochemicals added to the soil or substrate used for cultivation [11].

For the most part, the absorbent materials on the market are based on acrylate derivatives, such as crosslinked polyacrylic acids and copolymers of partial hydrolysis products of starches with acrylonitrile and starch–acid graft copolymers [11,12]. Their synthetic origin (derived from petroleum) implies that their biodegradability is low and, in some cases, they could be toxic for agricultural and human consumption purposes [13,14]. Crosslinking is one of the main techniques used for the fabrication of hydrogels, because it limits the mobility of the polymeric structure, reduces its solubility in water, and improves its mechanical and barrier properties [15]. Chemical crosslinking involves the formation of covalent bonds via irradiation, sulfur vulcanization, or chemical reactions. On the other hand, physical crosslinking uses non-covalent bonds, such as ionic interactions, hydrogen bridges, and hydrophobic interactions [16,17,18]. This is achieved through heating/cooling, freeze–thaw cycles, pH variations, and the interaction of polyelectrolytes and metal ions [19]. Physical hydrogels are inhomogeneous and present low levels of crosslinking; thus, their structural stability is very low [9,20]. However, the main advantage of physical crosslinking techniques lies in the fact that they do not require the use of any crosslinking agent, thus avoiding the toxicity problems commonly associated with this type of compound [19].

One of the solutions proposed to increase the biodegradability of absorbent materials used in agriculture is the replacement of petroleum-derived materials with natural polymers because of their excellent hydrophilic properties, abundance, and low cost [21]. However, the use of some of these biopolymers, such as starch, can affect food safety [22]. Therefore, much of the research in this area has been focused on the exploration of natural polymers with low food potential, such as alginate, chitosan, cellulose, and some proteins [8,23,24,25,26]. Among the proteins with the potential for the development of hydrogels is silk sericin (SS), composed mostly of polar amino acids with carboxyl and hydroxyl side chains [27]. This composition gives it a high water absorption capacity [28] and good barrier properties. Moreover, SS is thermally stable below 200 °C and exhibits a T_g_ between 170 °C and 197 °C [29,30]. However, SS-based materials are characterized by inferior mechanical properties to those found in petroleum-derived polymers [14]. Therefore, it is necessary to blend SS with other materials, such as polyvinyl alcohol (PVA) or glycerol [31,32,33,34,35,36], and/or crosslink it with glutaraldehyde, polyphenols, enzymes (TGase), carbodiimides (EDC), and oxidized polysaccharides [37,38,39,40,41,42,43,44,45,46]. The use of silk sericin materials has been evaluated mostly in pharmacological, cosmetic, and biomedical applications, and to a lesser extent, as heavy metal adsorbents and seed coatings [47,48,49]. However, its biodegradable nature, barrier properties, and ability to absorb large amounts of water [28,50] make it a promising material for use in agricultural production to make more efficient the use of water and nutrients in agricultural production.

Different methodologies have been used to evaluate the effect of hydrogels on the water retention capacity of the soils [51]. A significant number of investigations have studied, at the laboratory level, the use of this type of material in coarse, sandy, or sandy loam soil (SLS) [52]. Among the most commonly used methods for evaluating water retention capacity are the gravimetric method and Richard’s pressure plates [52,53,54,55]. Particularly, the gravimetric method can provide data that could give a better understanding of the dynamics of water content in the soil. Furthermore, these data can be used to create prediction models, which serve as building blocks for designing control systems for precision irrigation [56]. This makes it possible to have a better understanding of how hydrogels work as soil conditioners and can optimize agricultural production and water management.

The present study proposes the use of silk sericin in the development of novel hydrogels as a technological alternative to make more efficient use of water in agricultural production, and actually proposes and evaluates a new hydrogel-type material. The porous morphology of this new hydrogel was evaluated and correlated with changes in the water retention curve of a sandy loam soil (SLS). Additionally, changes in soil moisture dynamics upon incorporation of the hydrogel were studied by adjusting and analyzing different predictive models.

## 2. Materials and Methods

### 2.1. Materials

Defective cocoons (unreelable, double, stained, and pierced) of the Colombian hybrid *Bombyx mori* L., a byproduct of silk production in the region, were used as the main raw material in the hydrogel fabrication process. The cocoons were purchased from Corporación para el Desarrollo de la Sericultura del Cauca-CORSEDA (Popayán, Colombia). Polyvinyl alcohol PVA (Mw: 146,000–186,000) hydrolyzed 99%, purchased from Sigma-Aldrich (St. Louis, MO, USA), and food grade carboxymethyl cellulose (CMC, high viscosity 3500–4500 cP, degree of substitution: 0.7–0.9), acquired from Antioqueña de Químicos SAS (Medellín, Colombia), were used as additional inputs.

### 2.2. Fabrication of Hydrogels

For the extraction of sericin (SS), the degumming method, at high temperature and pressure in an autoclave, was used [29]. The silk cocoons were cut and subsequently immersed in distilled water, using a 1:30 ratio (g of cocoon: mL of water), and then degummed in an AVautoclave (Phoenix Luferco, Araraquara, Brazil) at 120 °C for 30 min. The resulting solution was concentrated by a freeze–thaw precipitation process to obtain a concentrated 2% *w*/*v* SS solution. In parallel, according with previously reported methodology [57], an aqueous solution of carboxymethylcellulose (CMC) and polyvinyl alcohol (PVA) at 1.25% *w*/*v* was prepared, to which concentrated SS solution was added to form a mixture of 45% SS, 25% CMC, and 30% PVA. The obtained mixture of SS/CMC/PVA (1.5% *w*/*v*) was deposited in plastic Petri dishes (90 mm in diameter) and left to stand overnight (18 h). Subsequently, the containers with the mixture were placed in a forced convection oven (Industrias Centricol, Medellín, Colombia) for 24 h at 40 °C to form hydrogel films (H). 

### 2.3. Morphological Characterization of the Hydrogel

The morphology of the hydrogels was studied using a Scanning Electron Microscope SEM, JEOL JSM-6490 LV (JEOL, Peabody, MA, USA), at a voltage of 15 kV. Prior to the analysis, the samples were prepared following the procedure described by Qiao et al. [58] to preserve the porous structure of the hydrogel. SEM images were analyzed using the open-source software ImageJ [59], from which 100 measurements of pore diameter were obtained to estimate 2 statistical parameters: average pore diameter and the value of the pore diameter below which 90% of the measures fall (90th percentile).

### 2.4. Soil

Soil from the municipality of Sopetrán, Colombia, (6°30′08.3″ N 75°44′15.2″ W) was used. For soil sample collection, the vegetation cover (branches, roots, and other vegetation remains) was removed, and samples were collected at a depth of 0–20 cm. According to the texture analysis performed using the Bouyoucos method [60], the soil sample obtained was of the sandy loam type (SLS), with relative sand, silt, and clay contents of 76%, 14%, and 10%, respectively. A total of 2 soil samples were evaluated in this study: soil without hydrogel (SLS) and soil mixed with 1% hydrogel powder (SLS + H), following previously reported methods [61,62,63]. For the homogeneous mixing of SLS with the hydrogel, soil with the hydrogel incorporated at a 1:100 (H: soil) mass ratio was placed in a plastic bucket (10 L) with a cover. Therefore, the bucket was rotated 20 times to obtain a homogenous sample.

### 2.5. Soil Moisture Retention Curves: Static Characterization

The Richards pressure chamber method was used to determine water retention in soil with and without hydrogel, given a matric potential (MP) or suction pressure, which is a measure of the energy required to remove water from soil pores due to the attractive forces between soil particles and water molecules [64]. Overall, 4 MP values were evaluated—0.3, 1, 5, and 15 bar—to determine the amount of water retained by each sample under characteristic suction conditions [65].

To use this method, 30 g (*W*_0_) of soil samples were taken, 1 for each MP point, which was saturated with water and then placed in a sealed chamber, following the methodology reported by Weil et al. [66]. During the test, each MP was simulated by injecting pressurized gas into the chamber. In this way, each sample was subjected to a specific pressure, and excess water was allowed to flow out, passing through a porous plate to leave the sealed chamber. When the sample reached equilibrium, which was achieved when the water flow stopped, it was removed from the equipment and weighed (*W_s_*). After drying in an oven at 105 °C for 48 h, the dry sample was weighed (*W_d_*) to determine gravimetric soil water/moisture content using Equation (1).

(1) $$Gravimetric\;soil\;water\;content\;\left(GWC\right)\;\left(\%\right)=\frac{W_{s}-W_{d}}{W_{0}}\times 100$$

### 2.6. Dynamic Soil Moisture Content Characteristics

To study soil moisture content dynamics, gravimetric monitoring was carried out on soil samples, with and without hydrogel, during the stages of saturation, accelerated drainage, slow drainage, and evaporation. Generally, the saturation stage is short and occurs between the moment when irrigation begins and the moment when water fills all the pores or empty spaces in the sample, up to the point when the sample cannot absorb any more moisture and water begins to leave the sample by gravity, which initiates the accelerated drainage stage. After a few hours, and for two or three days, a slow drainage rate is reached, after which the last stage is reached, in which the soil sample is at field capacity, and water loss occurs only by evaporation [66].

Dry samples were weighed in a container for monitoring. This initial weight is referred to as *W*_0_. A volume of 50 mL of water was added to each sample and reweighed (*W*_1_). The containers with the hydrated samples were kept at 40 °C and 50% relative humidity (RH) for 36 h using a DIES C115 climatic chamber (DIES, Itagüí, Colombia). The samples were weighed every 2 h (*W_i_*), and the gravimetric soil water content for each time *i* was calculated according to Equation (2):

(2) $$Gravimetric\;soil\;water\;content\;i\;\left(GWC_{i}\right)\;\left(\%\right)=\frac{W_{i}-W_{0}}{W_{1}-W_{0}}\times 100$$

The experiments were conducted five times (*n* = 5) at the Laboratory of the Agroindustrial Research Group (GRAIN) of the Universidad Pontificia Bolivariana (6°14′33″ N, 75°35′23″ W).

According to the observed behavior of the data obtained for the two treatments of interest, SLS and SLS + H, two types of models were fitted: one linear, with interval time-varying gain (piecewise linear model), and one dynamic of the first order. OriginPro 2016 software was used for the piecewise linear regression analysis. Initially, the slope at each point of the SLS and SLS + H curves was calculated using the first derivative of the data. Then, intervals determined by the lapses in which the slope of each curve remained approximately constant were defined. Thus, 3 sections were defined for each curve: from 0 to 2 h, associated with a stage of accelerated drainage; between 2 and 24 h, in which slow drainage and evaporation phenomena were combined; and finally, from 24 to 36 h. In the last stage, evaporation was only observed in the SLS + H sample, and the SLS sample was completely dry.

To fit the first-order dynamic model, the data were analyzed using the MATLAB “System Identification Toolbox” tool. In total, 3 different cases were considered for each treatment: full measurement range (0–36 h), slow drainage and evaporation (2–36 h), and evaporation only (18–36 h). The models were evaluated based on the following criteria: final prediction error (FPE), sum of squares of error (MSE), and coefficient of determination (R^2^) [56].

## 3. Results and Discussions

### 3.1. Morphological Characterization of Hydrogels

**Figure 1:**
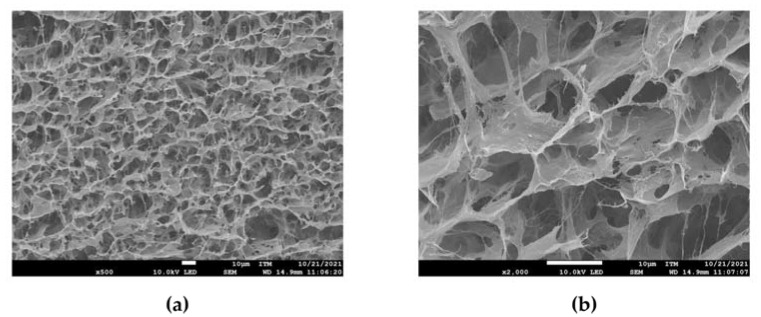
SEM images of 500× (**a**) and 2000× (**b**) of the hydrogel obtained from the SS/CMC/PVA mixture.

SEM micrographs of the SS/CMC/PVA hydrogel are shown in Figure 1. Notably, the processing method allows the formation of a porous structure. Physical crosslinking mechanisms, such as hydrogen bonding, electrostatic attractions, and ion–dipolar complexes, domain the formation of an interconnected pore structure [57,67,68]. Moreover, planar pore layers could be associated with interactions between SS and CMC that lead to the formation of SS sheet-like structures [69].

Analysis of the pore diameter distribution revealed that the average pore size was approximately 8 µm and that 90% of the pores had diameters equal to or smaller than 15 µm. This result suggests that a large part of the water that can be absorbed by the developed material could be used by plants, since plant roots have the capacity to obtain water present in pores with a diameter greater than 3 μm [66]. Accordingly with similar studies [70,71], the exhibited porous structure by the hydrogel and its polymer composition, which has a strong attraction to water, could also be associated with a good swelling behavior. Thus, it could be suggested that the SS/CMC/PVA composite hydrogel presented a suitable morphology for water retention in agricultural soils.

### 3.2. Soil Moisture Retention Curves: Static Characterization

**Figure 2:**
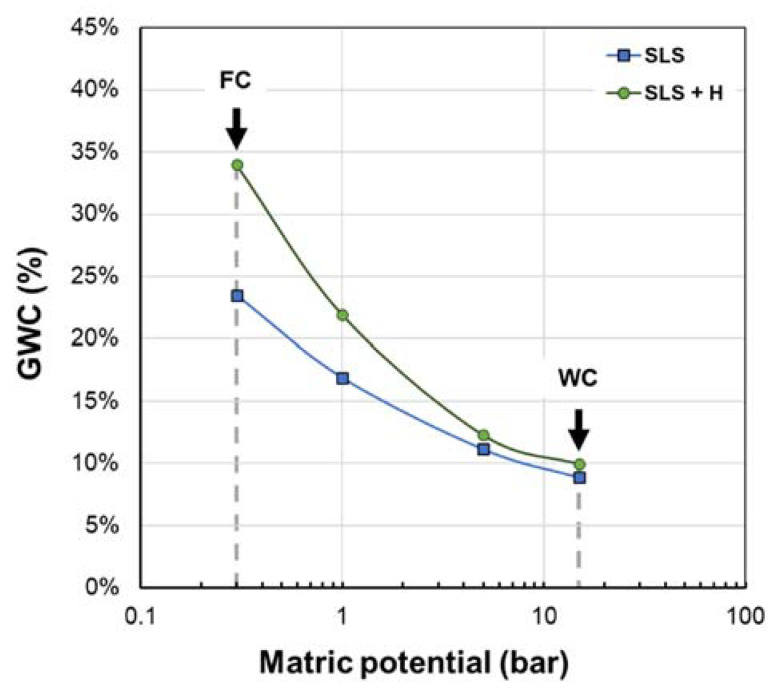
**Semilogarithmic** water retention curves for sandy loam soil (SLS) and sandy loam soil + hydrogel (SLS + H). GWC: gravimetric water content; FC: field capacity; WC: wilting coefficient.

The moisture retention curves obtained for the blank (SLS) and SLS + H are shown in Figure 2. These curves describe the relationship between the moisture content and soil matric potential, reflecting the capacity of the soil to retain water under different suction pressures. After saturation, a fraction of water is lost by drainage (gravitational water), while another fraction remains retained in soil micropores (plant-available water, PAW) or strongly adheres to soil particles (hygroscopic water) [66].

At a pressure of 0.3 bar, it was possible to determine the water retained in the soil after drainage, defined as the moisture at field capacity (FC). This measurement for the SLS + H was 33.98%, while for the blank, it was 23.49%, which indicates that the use of the hydrogel increased the moisture at the FC of the soil by 10.50%. Moreover, if the moisture at FC of SLS is used as a baseline, the percentage change of this parameter due to the incorporation of the hydrogel (SLS + H), calculated by Equation (3), is about 44.7%.

(3) $$Percentage\;change\;\left(\%\right)=\frac{\left(Moisture\;at\;\mathrm{FC}\;for\;\mathrm{SLS}+H\right)-\left(Moisture\;at\;\mathrm{FC}\;for\;\mathrm{SLS}\right)}{\left(Moisture\;at\;\mathrm{FC}\;for\;\mathrm{SLS}\right)}*100$$

This result is similar to those reported by Abhisekh et al. [52] and Agaba et al. [53], and it is associated with changes in the microstructure of soils. Hydrogels, when interacting with the water added to the soil, increase their size (swelling) until reaching equilibrium, thus acting as water reservoirs for the plants and at the same time altering the pore structure of the soil, i.e., the average diameter of the pores decreases, as well as the number of large pores. These changes in soil structure reduce gravitational water and promote water retention by capillarity [72].

By increasing the metric pressure up to 15 bar, all the PAW was subtracted; at this point, the hygroscopic water, also known as moisture at wilting coefficient (WC), was determined to be 9.95% for SLS + H and 8.86% for SLS. This suggests that much of the water absorbed by the hydrogel was effectively released, which is considered promising for its application as a moisture retainer in soils [52].

When analyzing the plant-available water (PAW), defined as the difference between the moisture at field capacity and the permanent wilting point [52], it was found that this indicator for SLS + H, with respect to the blank, increased by 9.40%. This finding demonstrates the potential of the developed material to improve agricultural water efficiency by improving water retention in the soil.

### 3.3. Dynamic Soil Moisture Content Characteristics

The effect of hydrogel incorporation on the water retention of the study soil was analyzed under extreme environmental conditions, such as those found in arid and semi-arid regions (40 °C, 50% RH). The possibility of using a hydrogel-type material is of particular interest because evapotranspiration tends to reduce water retention in soils, which, for agricultural production, results in a high demand for water resources.

**Figure 3:**
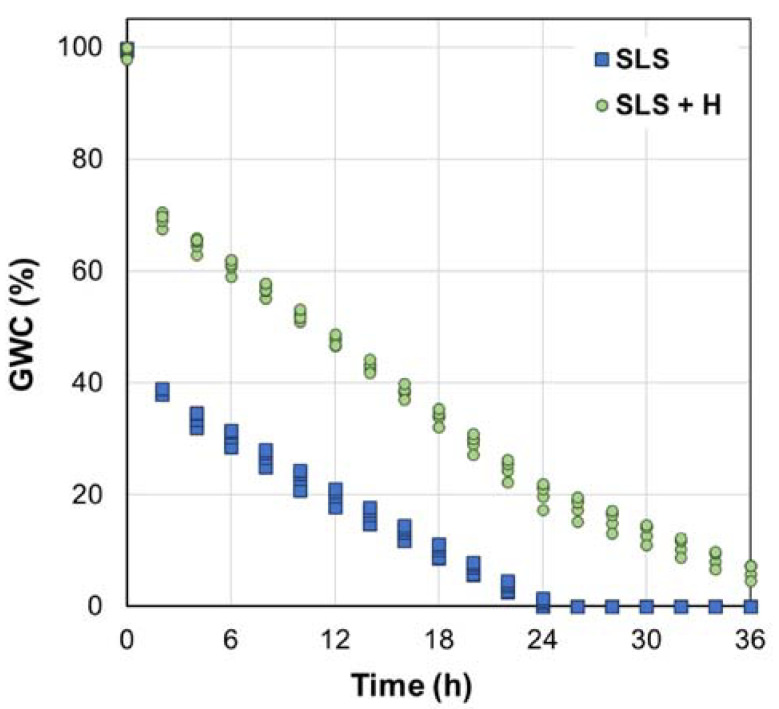
Soil moisture content data obtained at 40 °C and 50% RH. SLS: sandy loam soil; SLS + H: soil + hydrogel.

The moisture values obtained over time are shown in Figure 3. Immediately after irrigation (*t* = 0 h), the moisture content of both soils reached saturation, well above the field capacity, which was established at 24% for SLS and 34% for SLS + H. This excess moisture decreased rapidly at the beginning of the test owing to the drainage loss. After the first two hours, water loss was slower, with a much lower slope than that in the first segment. At 18 h, the drainage stopped, and water loss was mainly associated with redistribution and evaporation phenomena. Finally, after 24 h, the blank (SLS) showed 0% moisture, whereas the SLS + H retained 20% moisture. This finding confirms that the hydrogel has the capacity to improve water retention in the studied soil under the conditions evaluated.

In view of the nature of the behavior of gravimetric moisture over time, it was considered the possibility of complementing the morphological characterization of the sample using mathematical models, whose parameters could be adapted to future tests of this or other materials in different types of soil. The behavior of the data suggests that suitable models could be time-varying gain in sections and a first-order dynamic one.

The following sections are defined to fit the piecewise linear time (*t*)-varying gain model.

(4) $$\begin{array}{cc} 0\leq t\leq 2 & \\ 2<t\leq 24 & \\ t>24 & \end{array}$$

**Figure 4:**
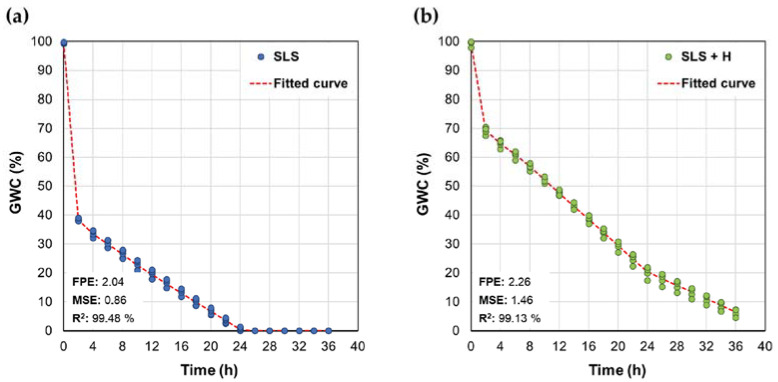
Fitted piecewise linear models for gravimetric soil water content data for (**a**) SLS and (**b**) SLS + H.

The fit results of this type of model for each case are presented in Table 1 and Figure 4.

When comparing the parameters by intervals, it is evident that, in the region associated with the accelerated drainage stage (0 ≤ *t* ≤ 2 h), the slope of the blank is much more negative than that found for the SLS + H system, suggesting that the presence of the hydrogel makes water loss less pronounced, due to the fact that it improves water retention in the soil [52]. Then, in the range between 2 and 24 h, the slopes were in the same range of magnitude for both treatments, suggesting that the evaporation phenomena of the remaining water after the accelerated drainage stage did not differ considerably between SLS and SLS + H. This, coupled with the fact that the SLS intercept in this range (≈40%) is much lower than that of the SLS + H system (~74%), indicates that the greatest impact of the hydrogel on water retention occurs in the drainage stage. Finally, for the system without hydrogel, the moisture value is considered constant and equal to zero; for the SLS + H system, this variable can be described by a linear function with a slight negative slope, suggesting the possibility of extending the test in time for future trials, so that it can be analyzed how long moisture persists.

**Figure 5:**
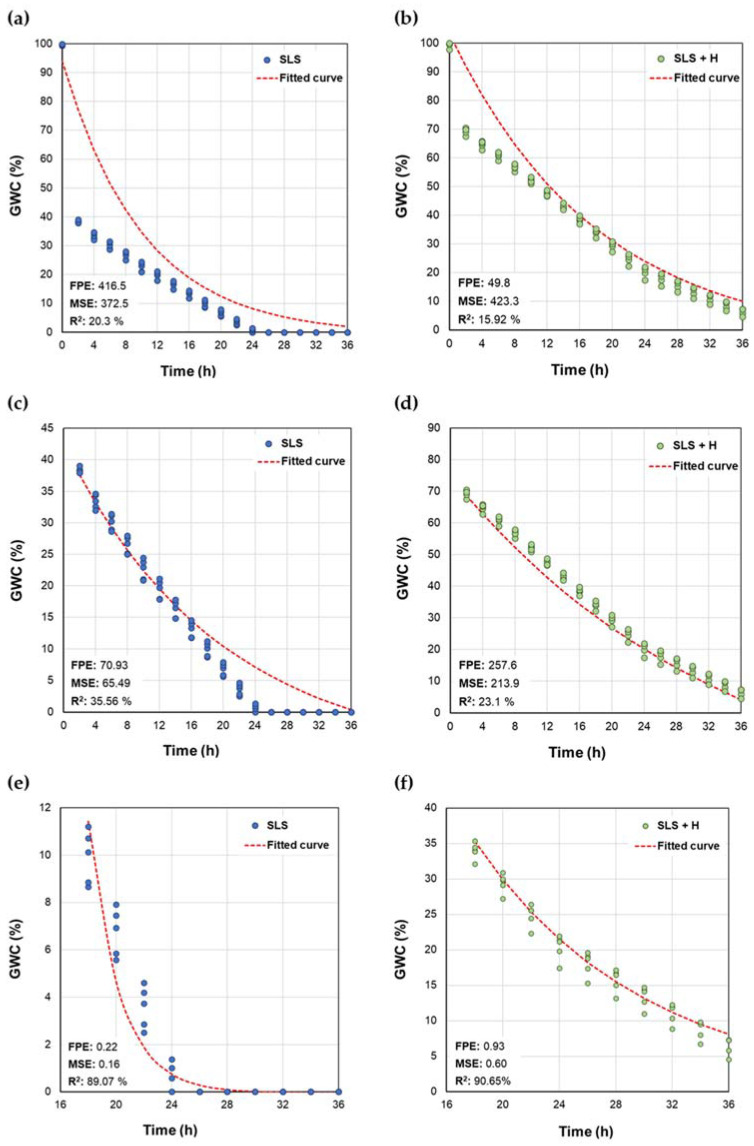
Data and fitted first-order transfer function for SLS and SLS + H in the intervals 0 ≤ *t* ≤ 36 h (**a**,**b**), 2 ≤ *t* ≤ 36 h (**c**,**d**), and 18 ≤ *t* ≤ 36 h (**e**,**f**).

The fit of the static model by section, as well as the statistical criteria used to evaluate it, are presented in Figure 5. In both cases, R^2^ values greater than 99% and low FPE and MSE values were obtained, indicating that this type of model adequately described the nature of the data. The first-order dynamic model represents the structure described by Equation (5):

(5) $$G\left(s\right)=\frac{K_{p}}{1+\tau_{p}}$$

In (4), *G*(*s*) is the transfer function name modeling the system, K_p_ the static gain of the system, and τ_p_ the time constant of the system in seconds. Models were obtained in this way using the MATLAB numerical identification tool for the full data set (0 ≤ *t* ≤ 36 h), for the fraction of the data associated with slow drainage (2 ≤ *t* ≤ 36 h), and for the evaporation stage (18 ≤ *t* ≤ 36 h). The resulting models for the different cases are listed in Table 2.

When analyzing the parameters of the models obtained, it was found that the time constant (τ_p_) for both cases of the SLS + H system was greater than that estimated for the systems without hydrogel. Accordingly, it can be affirmed that when the hydrogel is incorporated into SLS, more time is required to reach the steady state, which, in this case, is defined as the situation in which the soil presents a low moisture content, with little or no variability over time. From these results, it is possible to affirm that the adjusted transfer functions describe the impact of the absorbent material on the dynamics of the soil moisture content. The responses of the fitted models, as well as the statistical criteria used to evaluate their fit to the behavior of the data, are presented in Figure 5.

Among the adjusted models, with and without drainage, it is evident that in the latter case, more accurate models are obtained, in terms of higher R^2^ values, as well as a significant reduction in the FPE and MSE, with respect to the models based on data with drainage. This result confirms that the dynamics of water loss by drainage exhibit a different behavior than that developed during a stage in which the evaporation phenomenon predominates, and that this is more susceptible than drainage to be modeled by means of a first-order transfer function. This appreciation establishes clear conditions for the use of this type of model in the processes of simulation and automatic control of soil moisture.

In comparison with the piecewise linear model, the first-order model offers the advantage of not requiring updating of the gain over time or in the event of a change in the initial moisture level, if operating zones associated with the evaporation phenomenon are defined.

## 4. Conclusions

The new hydrogel developed, based on 70% natural polymers (SS/CMC), presented a desirable composition and structure for use as a material for water retention in soil. The porous structure observed in the SS/CMC/PVA hydrogel, as well as its affinity with water due to its composition, was associated with a good swelling behavior that was further reflected in the increase of the water retention capacity of SLS. By incorporating 1% hydrogel in the SLS, it was possible to increase the plant water available by 9.40%. This result suggests that hydrogel incorporation in SLS possibly changes the pore structure of SLS, promoting the formation of new micropores and, therefore, reducing the water loss by gravity.

This study explored the use of dynamic models to represent the behavior of moisture over time in each substrate to facilitate subsequent simulations or designs of automatic moisture control systems. Two mathematical models are proposed to describe the behavior of the data, one of which is piecewise linear, and the other is a first-order. Both models adequately fit this phenomenon. The linear curve can be found more directly and represents the behavior of water retention in the time domain; however, its gain changes by intervals. On the other hand, the first-order model requires identifying more parameters, but allows a description of the phenomenon in a wider dynamic region with a single fitted model.

For the linear model, the function sections for SLS and SLS + H were associated with the occurrence of accelerated drainage, slow drainage, and evaporation, which is reflected in the fact that the adjustment yielded a different gain for each section. When comparing the models by sections, it was found that in that associated with slow drainage (2 < *t* ≤ 24 h), the slopes do not present a significant difference, which may indicate that the phenomena of slow drainage and evaporation between these systems do not differ considerably. However, in the same section, it was observed that the SLS + H system presented a higher intercept (~74%) than that in the SLS (≈40%) in the slow drainage interval, suggesting that the hydrogel has a greater incidence over water retention in the accelerated drainage stage.

Regarding the first-order transfer function, the use of data that does not involve the drainage stage produces more accurate models because the dynamics of water in the soil differ between the drainage and evaporation stages. When analyzing the selected models, it was observed that the time constant of the SLS + H system was five times that found in SLS, demonstrating that the fitting of this type of model allows studying the impact of the absorbent material on the dynamics of soil moisture content. Altogether, the findings of this study indicate that the obtained hydrogel could be suitable as a material for soil water retention, being a promising strategy for lowering irrigation frequency in agricultural crops.

## Figures and Tables

**Table 1:** Estimated parameters for the adjusted piecewise linear models.

System	Model	
SLS	$GWC=\left\{\;\begin{array}{c} -30.657t+99.631\\ -1.6807t+40.219\\ 0 \end{array}\right.$	0≤t≤2 2<t≤24 t>24
SLS + H	$GWC=\left\{\;\begin{array}{c} -14.968t+99.377\\ -2.238t+74.245\\ -1.1595t+48.172 \end{array}\right.$	0≤t≤2 2<t≤24 t>24

**Table 2:** Estimated parameters for first-order transfer functions.

System	Interval	Static Gain K_p_	Time Constant τ_p_
SLS	0 ≤ *t* ≤ 36 h	2.3692	36,551
	2 ≤ *t* ≤ 36 h	1.9989	70,060
	18 ≤ *t* ≤ 36 h	0.4910	8110
SLS + H	0 ≤ *t* ≤ 36 h	7.2242	65,466
	2 ≤ *t* ≤ 36 h	6.4081	124,010
	18 ≤ *t* ≤ 36 h	0.11657	43,098

## Data Availability

The data presented in this study are available upon request from the corresponding author.

## References

[1] United Nations, Department of Economic and Social Affairs (2019). World Population Prospects 2019—Highlights (ST/ESA/SER.A/423).

[2] Li S., Chen G. (2019). Contemporary Strategies for Enhancing Nitrogen Retention and Mitigating Nitrous Oxide Emission in Agricultural Soils: Present and Future. Environ. Dev. Sustain..

[3] Organización de las Naciones Unidas para la Alimentación y la Agricultura (FAO) La Agricultura Mundial En La Perspectiva Del Año 2050. Proceedings of the Cómo Alimentar al Mundo 2050.

[4] Drescher A., Glaser R., Richert C., Nippes K.R. (2011). Demand for Key Nutrients (NPK) in the Year 2050.

[5] Daneshgar S., Callegari A., Capodaglio A., Vaccari D. (2018). The Potential Phosphorus Crisis: Resource Conservation and Possible Escape Technologies: A Review. Resources.

[6] Campos E., de Oliveira J.L., Fraceto L.F., Singh B. (2014). Polysaccharides as Safer Release Systems for Agrochemicals. Agron. Sustain. Dev..

[7] Lenka S., Lenka N.K., Singh A.B., Singh B., Raghuwanshi J. (2017). Global Warming Potential and Greenhouse Gas Emission under Different Soil Nutrient Management Practices in Soybean–Wheat System of Central India. Environ. Sci. Pollut. Res..

[8] Feng D., Bai B., Wang H., Suo Y. (2017). Novel Fabrication of Biodegradable Superabsorbent Microspheres with Diffusion Barrier through Thermo-Chemical Modification and Their Potential Agriculture Applications for Water Holding and Sustained Release of Fertilizer. J. Agric. Food Chem..

[9] Hoffman A. (2012). Hydrogels for Biomedical Applications. Adv. Drug Deliv. Rev..

[10] Chen Y., Chen Y. (2020). Properties and Development of Hydrogels. Hydrogels Based on Natural Polymers.

[11] Ramli R. (2019). Slow Release Fertilizer Hydrogels: A Review. Polym. Chem..

[12] Ekebafe L., Ogbeifun D.E., Okieimen F.E. (2011). Polymer Applications in Agriculture. Biokemistri.

[13] Demitri C., Scalera F., Madaghiele M., Sannino A., Maffezzoli A. (2013). Potential of Cellulose-Based Superabsorbent Hydrogels as Water Reservoir in Agriculture. Int. J. Polym. Sci..

[14] Azeredo H., Waldron K. (2016). Crosslinking in Polysaccharide and Protein Films and Coatings for Food Contact—A Review. Trends Food Sci. Technol..

[15] Serrano-Aroca Á. (2018). Enhancement of Hydrogels Properties for Biomedical Applications: Latest Achievements. Hydrogels.

[16] Balaguer M.P., Gómez-Estaca J., Gavara R., Hernandez-Munoz P. (2011). Functional Properties of Bioplastics Made from Wheat Gliadins Modified with Cinnamaldehyde. J. Agric. Food Chem..

[17] Hennink W.E., van Nostrum C.F. (2012). Novel Crosslinking Methods to Design Hydrogels. Adv. Drug Deliv. Rev..

[18] Hu H., Xu F. (2020). Rational Design and Latest Advances of Polysaccharide-Based Hydrogels for Wound Healing. Biomater. Sci..

[19] Oryan A., Kamali A., Moshiri A., Baharvand H., Daemi H. (2018). Chemical Crosslinking of Biopolymeric Scaffolds: Current Knowledge and Future Directions of Crosslinked Engineered Bone Scaffolds. Int. J. Biol. Macromol..

[20] Guilherme M., Aouada F.A., Fajardo A.R., Martins A.F., Paulino A.T., Davi M.F.T., Rubira A.F., Muniz E.C. (2015). Superabsorbent Hydrogels Based on Polysaccharides for Application in Agriculture as Soil Conditioner and Nutrient Carrier: A Review. Eur. Polym. J..

[21] Luo M.T., Li H.L., Huang C., Zhang H.R., Xiong L., Chen X.F., Chen X. (2018). De Cellulose-Based Absorbent Production from Bacterial Cellulose and Acrylic Acid: Synthesis and Performance. Polymers.

[22] Skrzypczak D., Witek-Krowiak A., Dawiec-Liśniewska A., Podstawczyk D., Mikula K., Chojnacka K. (2019). Immobilization of Biosorbent in Hydrogel as a New Environmentally Friendly Fertilizer for Micronutrients Delivery. J. Clean. Prod..

[23] Kong W., Li Q., Li X., Su Y., Yue Q., Gao B. (2019). A Biodegradable Biomass-Based Polymeric Composite for Slow Release and Water Retention. J. Environ. Manag..

[24] Calabria L., Vieceli N., Bianchi O., Boff de Oliveira R.V., do Nascimento Filho I., Schmidt V. (2012). Soy Protein Isolate/Poly(Lactic Acid) Injection-Molded Biodegradable Blends for Slow Release of Fertilizers. Ind. Crops Prod..

[25] Pushpamalar J., Langford S.J., Ahmad M.B., Lim Y.Y., Hashim K. (2017). Eco-Friendly Smart Hydrogels for Soil Conditioning and Sustain Release Fertilizer. Int. J. Environ. Sci. Technol..

[26] Zhang Y.-Q. (2002). Applications of Natural Silk Protein Sericin in Biomaterials. Biotechnol. Adv..

[27] Gupta D., Agrawal A., Chaudhary H., Gulrajani M., Gupta C. (2013). Cleaner Process for Extraction of Sericin Using Infrared. J. Clean. Prod..

[28] Jaramillo-Quiceno N., Callone E., Dirè S., Álvarez-López C., Motta A. (2021). Boosting Sericin Extraction through Alternative Silk Sources. Polym. J..

[29] Nakamura S., Magoshi J., Magoshi Y., Kaplan D., Adams W., Farmer B., Viney C. (1993). Thermal Properties of Silk Proteins in Silkworms. Silk Polymers.

[30] Aramwit P., Ratanavaraporn J., Ekgasit S., Tongsakul D., Bang N. (2015). A Green Salt-Leaching Technique to Produce Sericin/PVA/Glycerin Scaffolds with Distinguished Characteristics for Wound-Dressing Applications. J. Biomed. Mater. Res. Part B Appl. Biomater..

[31] Lim K.S. (2014). Fabrication and Characterisation of Degradable Biosynthetic Hydrogels for Cell Encapsulation: Development of a New Method for Protein Incorporation. Ph.D. Thesis.

[32] Zhang Y., Zhao Y., He X., Fang A., Jiang R., Wu T., Chen H., Cao X., Liang P., Xia D. (2019). A Sterile Self-Assembled Sericin Hydrogel via a Simple Two-Step Process. Polym. Test..

[33] Tao G., Wang Y., Cai R., Chang H., Song K., Zuo H., Zhao P., Xia Q., He H. (2019). Design and Performance of Sericin/Poly(Vinyl Alcohol) Hydrogel as a Drug Delivery Carrier for Potential Wound Dressing Application. Mater. Sci. Eng. C.

[34] Zhang H., Deng L., Yang M., Min S., Yang L., Zhu L. (2011). Enhancing Effect of Glycerol on the Tensile Properties of Bombyx Mori Cocoon Sericin Films. Int. J. Mol. Sci..

[35] Sothornvit R., Chollakup R., Suwanruji P. (2010). Extracted Sericin from Silk Waste for Film Formation. Songklanakarin J. Sci. Technol..

[36] Wang K., Zhan F. (2017). Preparation and Properties of Silk Sericin/Cellulose Cross-Linking Films. MATEC Web Conf..

[37] Purwar R., Verma A., Batra R. (2019). Antimicrobial Gelatin/Sericin/Clay Films for Packaging of Hygiene Products. J. Polym. Eng..

[38] Boonpavanitchakul K., Pimpha N., Kangwansupamonkon W., Magaraphan R. (2020). Processing and Antibacterial Application of Biodegradable Sponge Nano-Composite Materials of Silver Nanoparticles and Silk Sericin. Eur. Polym. J..

[39] De Freitas E.D., Lima B.M., Rosa P.C.P., da Silva M.G.C., Vieira M.G.A. (2019). Evaluation of Proanthocyanidin-Crosslinked Sericin/Alginate Blend for Ketoprofen Extended Release. Adv. Powder Technol..

[40] das Graças Santos N.T., Landers R., da Silva M.G.C., Vieira M.G.A. (2020). Adsorption of Gold Ions onto Sericin and Alginate Particles Chemically Crosslinked by Proanthocyanidins: A Complete Fixed-Bed Column Study. Ind. Eng. Chem. Res..

[41] das Graças Santos N.T., Moraes L.F., da Silva M.G.C., Vieira M.G.A. (2020). Recovery of Gold through Adsorption onto Sericin and Alginate Particles Chemically Crosslinked by Proanthocyanidins. J. Clean. Prod..

[42] Guo X., Zhou Q., Wang P., Yu Y., Yuan J., Wang Q. (2020). Enzymatic Crosslinking of Silk Sericin through Combined Use of TGase and the Custom Peptide. J. Text. Inst..

[43] Ghensi P., Bettio E., Maniglio D., Bonomi E., Piccoli F., Gross S., Caciagli P., Segata N., Nollo G., Tessarolo F. (2019). Dental Implants with Anti-Biofilm Properties: A Pilot Study for Developing a New Sericin-Based Coating. Materials.

[44] Gallo N., Lunetti P., Bettini S., Barca A., Madaghiele M., Valli L., Capobianco L., Sannino A., Salvatore L. (2021). Assessment of Physico-Chemical and Biological Properties of Sericin-Collagen Substrates for PNS Regeneration. Int. J. Polym. Mater. Polym. Biomater..

[45] Wang P., He H., Cai R., Tao G., Yang M., Zuo H., Umar A., Wang Y. (2019). Cross-Linking of Dialdehyde Carboxymethyl Cellulose with Silk Sericin to Reinforce Sericin Film for Potential Biomedical Application. Carbohydr. Polym..

[46] Da Silva T.L., Da Silva A.C., Vieira M.G.A., Gimenes M.L., da Silva M.G.C. (2016). Biosorption Study of Copper and Zinc by Particles Produced from Silk Sericin—Alginate Blend: Evaluation of Blend Proportion and Thermal Cross-Linking Process in Particles Production. J. Clean. Prod..

[47] Kwak H.W., Yang Y.S., Kim M.K., Lee J.Y., Yun H., Kim M.H., Lee K.H. (2013). Chromium(VI) Adsorption Behavior of Silk Sericin Beads. Int. J. Ind. Entomol..

[48] Sonjan S., Ross G.M., Mahasaranon S., Sinkangam B., Intanon S., Ross S. (2021). Biodegradable Hydrophilic Film of Crosslinked PVA/Silk Sericin for Seed Coating: The Effect of Crosslinker Loading and Polymer Concentration. J. Polym. Environ..

[49] Wu J.-H.H., Wang Z., Xu S.-Y.Y. (2007). Preparation and Characterization of Sericin Powder Extracted from Silk Industry Wastewater. Food Chem..

[50] Werdin J., Fletcher T.D., Rayner J.P., Williams N.S.G., Farrell C. (2020). Biochar Made from Low Density Wood Has Greater Plant Available Water than Biochar Made from High Density Wood. Sci. Total Environ..

[51] Saha A., Rattan B., Sekharan S., Manna U. (2020). Quantifying the Interactive Effect of Water Absorbing Polymer (WAP)-Soil Texture on Plant Available Water Content and Irrigation Frequency. Geoderma.

[52] Agaba H., Baguma Orikiriza L.J., Osoto Esegu J.F., Obua J., Kabasa J.D., Hüttermann A. (2010). Effects of Hydrogel Amendment to Different Soils on Plant Available Water and Survival of Trees under Drought Conditions. CLEAN Soil Air Water.

[53] Koupai J.A., Eslamian S.S., Kazemi J.A. (2008). Enhancing the Available Water Content in Unsaturated Soil Zone Using Hydrogel, to Improve Plant Growth Indices. Ecohydrol. Hydrobiol..

[54] Saha A., Sekharan S., Manna U. (2020). Superabsorbent Hydrogel (SAH) as a Soil Amendment for Drought Management: A Review. Soil Tillage Res..

[55] Abioye E.A., Abidin M.S.Z., Mahmud M.S.A., Buyamin S., AbdRahman M.K.I., Otuoze A.O., Ramli M.S.A., Ijike O.D. (2021). IoT-Based Monitoring and Data-Driven Modelling of Drip Irrigation System for Mustard Leaf Cultivation Experiment. Inf. Process. Agric..

[56] Jaramillo-Quiceno N., Rueda-Mira S., Marín J.F.S., Álvarez-López C. (2023). Development of a Novel Silk Sericin-Based Hydrogel Film by Mixture Design. J. Polym. Res..

[57] Qiao D., Liu H., Yu L., Bao X., Simon G.P., Petinakis E., Chen L. (2016). Preparation and Characterization of Slow-Release Fertilizer Encapsulated by Starch-Based Superabsorbent Polymer. Carbohydr. Polym..

[58] Schneider C.A., Rasband W.S., Eliceiri K.W. (2012). NIH Image to ImageJ: 25 Years of Image Analysis. Nat. Methods.

[59] Jaramillo D.F. (2002). Introducción a la Ciencia del Suelo.

[60] Tanan W., Panichpakdee J., Suwanakood P., Saengsuwan S. (2021). Biodegradable Hydrogels of Cassava Starch-g-Polyacrylic Acid/Natural Rubber/Polyvinyl Alcohol as Environmentally Friendly and Highly Efficient Coating Material for Slow-Release Urea Fertilizers. J. Ind. Eng. Chem..

[61] Chen Y., Li W., Zhang S. (2021). A Multifunctional Eco-Friendly Fertilizer Used Keratin-Based Superabsorbent as Coatings for Slow-Release Urea and Remediation of Contaminated Soil. Prog. Org. Coatings.

[62] Wang W., Yang S., Zhang A., Yang Z. (2021). Synthesis of a Slow-Release Fertilizer Composite Derived from Waste Straw That Improves Water Retention and Agricultural Yield. Sci. Total Environ..

[63] Braudeau E., Hovhannissian G., Assi A.T., Mohtar R.H. (2014). Soil Water Thermodynamic to Unify Water Retention Curve by Pressure Plates and Tensiometer. Front. Earth Sci..

[64] Richards L.A. (1949). Methods of Measuring Soil Moisture Tension. Soil Sci..

[65] Weil R.R., Brady N.C., Fox D. (2017). The Nature and Properties of Soils.

[66] Xiao C., Gao Y. (2008). Preparation and Properties of Physically Crosslinked Sodium Carboxymethylcellulose/Poly(Vinyl Alcohol) Complex Hydrogels. J. Appl. Polym. Sci..

[67] Aramwit P., Sereemaspun A., Yamdech R. (2018). Sericin Ameliorates the Properties of Poly(Vinyl Alcohol) Hydrogel Prepared by Simple Repeated Freeze-Thaw Process without the Use of Chemical Crosslinking. Int. J. Res. Sci..

[68] Lin X., Jin J., Guo X., Jia X. (2021). All-Carboxymethyl Cellulose Sponges for Removal of Heavy Metal Ions. Cellulose.

[69] Mazloom N., Khorassani R., Zohuri G.H., Emami H., Whalen J. (2019). Development and Characterization of Lignin-Based Hydrogel for Use in Agricultural Soils: Preliminary Evidence. CLEAN Soil Air Water.

[70] El-Rehim H.A.A., Hegazy E.-S.A., El-Mohdy H.L.A. (2004). Radiation Synthesis of Hydrogels to Enhance Sandy Soils Water Retention and Increase Plant Performance. J. Appl. Polym. Sci..

[71] Narjary B., Aggarwal P., Singh A., Chakraborty D., Singh R. (2012). Water Availability in Different Soils in Relation to Hydrogel Application. Geoderma.

[72] Zotarelli L., Dukes M.D., Morgan K.T. (2013). Interpretación Del Contenido de La Humedad Del Suelo Para Determinar Capacidad de Campo y Evitar Riego Excesivo En Suelos Arenosos Utilizando Sensores de Humedad. Edis.

